# Long-term *in vitro* culture and preliminary establishment of chicken primordial germ cell lines

**DOI:** 10.1371/journal.pone.0196459

**Published:** 2018-04-30

**Authors:** Linglin Kong, Lingling Qiu, Qixin Guo, Ying Chen, Xin Zhang, Bowen Chen, Yang Zhang, Guobin Chang

**Affiliations:** College of Animal Science and Technology, Yangzhou University, Yangzhou, Jiangsu, P.R. China; Qingdao Agricultural University, CHINA

## Abstract

Primordial germ cells (PGCs) are precursors of functional gametes and can be used as efficient transgenic tools and carriers in bioreactors. Few methods for long-term culture of PGCs are available. In this study, we tested various culture conditions for PGCs, and used the optimum culture system to culture chicken gonad PGCs for about three hundred days. Long-term-cultured PGCs were detected and characterized by karyotype analysis, immunocytochemical staining of SSEA-1, c-kit, Sox2, cDAZL, and quantitative RT-PCR for specific genes like *Tert*, *DAZL*, *POUV*, and *NANOG*. Cultured PGCs labeled with PKH26 were reinjected into Stage X recipient embryos and into the dorsal aorta of Stage 14–17 embryos to assay their ability of migration into the germinal crescent and gonads, respectively. In conclusion, the most suitable culture system for PGCs is as follows: feeder layer cells treated with 20 μg/mL mitomycin C for 2 hours, and with 50% conditioned medium added to the factor culture medium. PGCs cultured in this system retain their pluripotency and the unique ability of migration without transformation, indicating the successful preliminary establishment of chicken primordial germ cell lines and these PGCs can be considered for use as carriers in transgenic bioreactors.

## Introduction

Due to the particularity of embryonic development, it is highly difficult to obtain gametes and single cell fertilized eggs from poultry. Primordial germ cells (PGCs), as precursors of functional gametes, demonstrate unique pluripotency and differentiation potential, as well as migration patterns during development, which are extremely different from those in mammals [1]. During early development, PGCs circulate temporarily in the bloodstream at the Hamburger and Hamilton (H&H) Stages 13–15, then migrate to the germinal ridges at Stage 27, and ultimately differentiate into oocytes and spermatocytes [2,3]. The unique migration pattern of chicken PGCs facilitates their isolation, which is ideal for the establishment of embryonic stem cell lines.

PGCs with multi-potential properties can be widely used in many fields such as germ cell and developmental biology, transgenesis and genome editing, conservation of avian genetic resources, and transgenic chicken production [4–9]. The prerequisite and basis of each field mentioned above is to establish and maintain PGC lines with multiple differentiation potentials and normal diploid karyotypes. However, several problems such as low proliferation rates with normal karyotype and the low number of cells involved in germline formation still exist in the long-term culture and establishment of PGC lines (van de Lavoir et al., 2006) [10]. Significant efforts have been made to solve these problems and it has been verified that chicken PGCs can be maintained *in vitro* without losing their properties in contrast to mammalian PGCs [7].

With the combination of growth factors, conditioned medium, and feeder cell layers, PGCs can maintain their proliferation rate and undifferentiated state in the long-term. Creating a suitable culture system for PGCs can facilitate their availability *in vitro* and retain their unique pluripotency and differentiation potential. The system can allow the use of PGCs as carriers in transgenic bioreactors, and as an ideal model for the study of transgenic chickens. Transgenic chickens will become an efficient system for supplying a tremendous amount of bioactive material to the development of functional foods and human pharmaceutics [11]. Therefore, establishing the PGCs culture system is an effective way for avian genetic engineering and protection of endangered poultry species.

This study aimed to investigate robust methods allowing the isolation and long-term culture of chicken gonadal PGCs, and to determine whether the system might alter their initial biological characterization or their gonadal colonization ability.

## Materials and methods

### PGC isolation, purification, and culture

All animal experimental procedures were approved and guided by the Institutional Animal Care and Use Committee of the School of Animal Science and Technology, Yangzhou University (Permit Number: 45, Government of Jiangsu Province, China) and the U.S. National Institute of Health guidelines (NIH Pub. No. 85–23, revised 1996).

Fertilized eggs (line White Leghorns; Yangzhou Ruinong Technology Co., Ltd) were incubated at 37.8°C and 70% relative humidity. After incubation for 120 h, PGCs were isolated from the embryonic gonads at Stage 27 with sharp forceps under a stereomicroscope and separated from red blood cells and chicken embryo fibroblasts by the differential adhesion method.

PGCs were placed on a feeder layer of mitomycin C-treated STO (SIM mouse *(Mus nusculus)* embryo-derived thioguanine and ouabain resistant) (This cell line was provided by professor Song CY. Yangzhou University, China) cells and cultured in an incubator at 37°C with 5% atmospheric CO_2_ and 60–70% relative humidity. The culture medium comprised knockout Dulbecco’s modified Eagle’s medium (KO-DMEM) (Gibco, NY, USA) supplemented with 10% fetal bovine serum (FBS) (Gibco), 2.5% chicken serum (CS) (Gibco), 2 mM L-glutamine (Gibco), 1× MEM nonessential amino acids (Gibco), 1× Nucleosides (Sigma Aldrich, St Louis, MO, USA), 1× HEPES (Gibco), 0.1 mM β-mercaptoethanol (Sigma Aldrich), 5 ng/mL human stem cell factor (hSCF) (Sigma Aldrich), 10 ng/mL basic fibroblast growth factor (bFGF) (Sigma Aldrich), and 10 ng/mL mouse leukemia inhibitory factor (LIF) (Sigma Aldrich). For subculture, the medium of cultured PGCs was changed every day, the cells dissociated using Accutase (Millipore, Billerica, MA, USA), and passaged on newly-treated feeder cells.

### Treatment of feeder layer

STO cells were cultured in Dulbecco’s modified Eagle’s medium (DMEM) (Hyclone, Logan, Utah, USA) containing 10% FBS (Gibco) in an incubator. To detect the optimum concentration of mitomycin C for feeder cell treatment, we treated STO cells with various concentrations of mitomycin C (10, 15, 20, and 30 μg/mL) for 2, 3, and 4 h. When the confluence reached 60%, cell viability was determined using the CCK-8 assay. Inactivated STO cells can be used as feeder layers for PGCs after subculture on complete medium for at least 24 h to prevent excessive toxicity of mitomycin C.

### Preparation of conditioned medium

BRL (Buffalo rat liver) (This cell line was provided by professor Song CY. Yangzhou University, China) cells were cultured in KO-DMEM (Gibco) containing 10% FBS (Gibco) and 2 mM L-glutamine (Gibco). The medium was changed in 24 h to removes the debris and waste from cells after passage. The conditioned medium was collected at 2 days after the medium change and was filtered before supplementing the medium for PGCs. To detect the optimum appending proportion of BRL conditioned medium, we supplemented the PGCs with 0%, 20%, 40%, 50%, 60%, and 80% of conditioned medium, and determined the viability of cultured PGCs using the CCK-8 assay. The BRL conditioned medium was stored at -80°C before use.

### Cryopreservation and thawing of PGCs

Cultured PGCs were harvested and resuspended in a cryoprotectant solution containing 10% dimethylsulfoxide (DMSO) (Gibco) and 90% FBS (Gibco). For cryopreservation, 2 × 10^6^–1 × 10^7^ cells were added to each cryogenic vial, placed in a freezing container with isopropanol, and cooled overnight in a -80°C freezer. Samples in cryogenic vials were warmed in a water bath at 37°C and oscillated until the disappearance of ice crystals. The cryoprotectant solution was then diluted 10-fold, immediately within 1 min.

### Cell viability detection

To evaluate cell viability, the Cell Counting Kit-8 (CCK-8) (Vazayme, Nanjing, China) was used according to the manufacturer’s instructions. Cells were seeded into 96-well plates and cell viability was detected by adding CCK-8 (10 μL) to each well, then incubating for 1.5 h. The resulting absorbance was measured at 450 nm on a microplate reader. The assay was repeated 3 times. Cell viability was calculated according to the following formula:

Cellviability=(ODofcontrol−ODoftreatment)/(ODofcontrol−ODofblank)×100%.

### Immunofluorescence assay

Cultured PGCs were seeded into a 24-well plate with a treated feeder layer and incubated for about 24 h. The cells were fixed in freshly prepared 4% paraformaldehyde-PBS for 20 min, then treated with a permeabilization buffer containing 0.5% Triton X-100 for 20 min. After washing thrice with PBS, the cells were blocked for 30 min and then incubated overnight with primary antibodies diluted to 5–20 μg/mL in BSA in the dark at 4°C. Primary antibodies against SSEA-1, c-kit, Sox2, cDAZL were used. Cells were incubated with fluorochrome-conjugated secondary antibodies for 1 h at room temperature (25°C). Cells were finally counter stained with DAPI (BOSTER, Wuhan, China) for 10 min, and analyzed under a fluorescence microscope.

### Cell cycle analysis

To analyze the cell cycle progression and apoptosis in fresh PGCs, thawed PGCs, and long-term cultured PGCs, the Cell Cycle and Apoptosis Analysis Kit (Beyotime, Jiangsu, China) was used according to the manufacturer’s instructions. Harvested cells were fixed overnight with 70% ethanol and incubated with RNase for 5 min. After addition of propidium iodide (PI) and incubating in the dark for 30 min at 37°C, DNA content was analyzed using a FACS Calibur flow cytometer within 24 hours.

### Soft agar assay

Thawed agar (1.2%) was cooled to 37°C and mixed with 2× DMEM containing 20% fetal bovine serum as the basal layer, to obtain a final agarose final concentration of 0.6%. Cells (1000 cells/mL) suspended in 0.3% agarose were then seeded onto the basal layer. A proper amount of medium was added to maintain moisture. Colony formation was observed microscopically for up to 7 days, and visible colonies were stained with crystal violet and counted under a stereoscopic microscope.

### Quantitative RT-PCR analysis

Total RNA was extracted from cultured PGCs (cultured for 15, 68, 180, and 268 days and thawed) using TRIzol reagent (Tiangen, Beijing, China). The RNA (1 μg) isolated for each condition was reverse transcribed to complementary DNA (cDNA) using the FastQuant RT Kit (with gDNase) (Tiangen). Target cDNA was amplified by quantitative PCR (qRT-PCR) using the primers specified in S1 Table. Gene expression levels were normalized against GAPDH expression. The experiment was performed on an ABI 7500 Real-time PCR Detection System (Applied Biosystems, Carlsbad, CA) using the UltraSYBR Mixture (with ROX) (CoWin Biotechnology, Beijing, China), and each sample was analyzed in triplicate.

### Karyotype analysis

Cultured PGCs were incubated with colcemid (final concentration 0.25 μg/mL) for 4 h at 37°C in 5% CO_2_, harvested, centrifuged, and resuspended in 0.075 M KCl prewarmed to 37°C. After incubation for 15 min at 37°C, the cells were pelleted again, fixed with 3:1 methanol:acetic acid, and washed thrice with the fixative. Finally, the cell suspension was dropped onto chilled glass slides and stained with Giemsa (Amresco, Solon, OH, USA). At least 20 metaphase spreads were counted for every chicken PGC passage.

### Gonadal migration assay

Long-term cultured PGCs were used for the migration assay. To assay migration into the germinal crescent, cells were labeled with PKH26 using the PKH26 Red Fluorescent Cell Linker Mini Kit (Sigma) and 3000 cells per egg were transferred into the subgerminal cavity described by Eyal-Giladi and Kochav [12] in Stage X recipient embryos. Recipient eggs sealed with Parafilm were further incubated for 24 h until Stage 6. The germinal crescent of excised embryos was observed under a fluorescence microscope. PKH26-labeled PGCs were also injected into the dorsal aorta of Stage 14–17 embryos to assay their migration into gonads. After sealing with Parafilm, these eggs were further incubated until Stage 30, and the gonads were observed under a fluorescence microscope.

### Retinoic acid induction assay

The cultured PGCs were to differentiated into germ cells using all-trans-retinoic acid (RA) (Sigma) to determine their differentiation potential. The PGCs were cultured in medium containing 2 μM/mL of RA without a feeder layer for 8 days, using the culture medium composition listed in S2 Table. Morphological changes were observed under an inverted microscope and total RNA was extracted and analyzed by quantitative RT-PCR after every 2 days.

## Results

### PGCs can be cultured in a compound culture system for a long time

**Fig 1:**
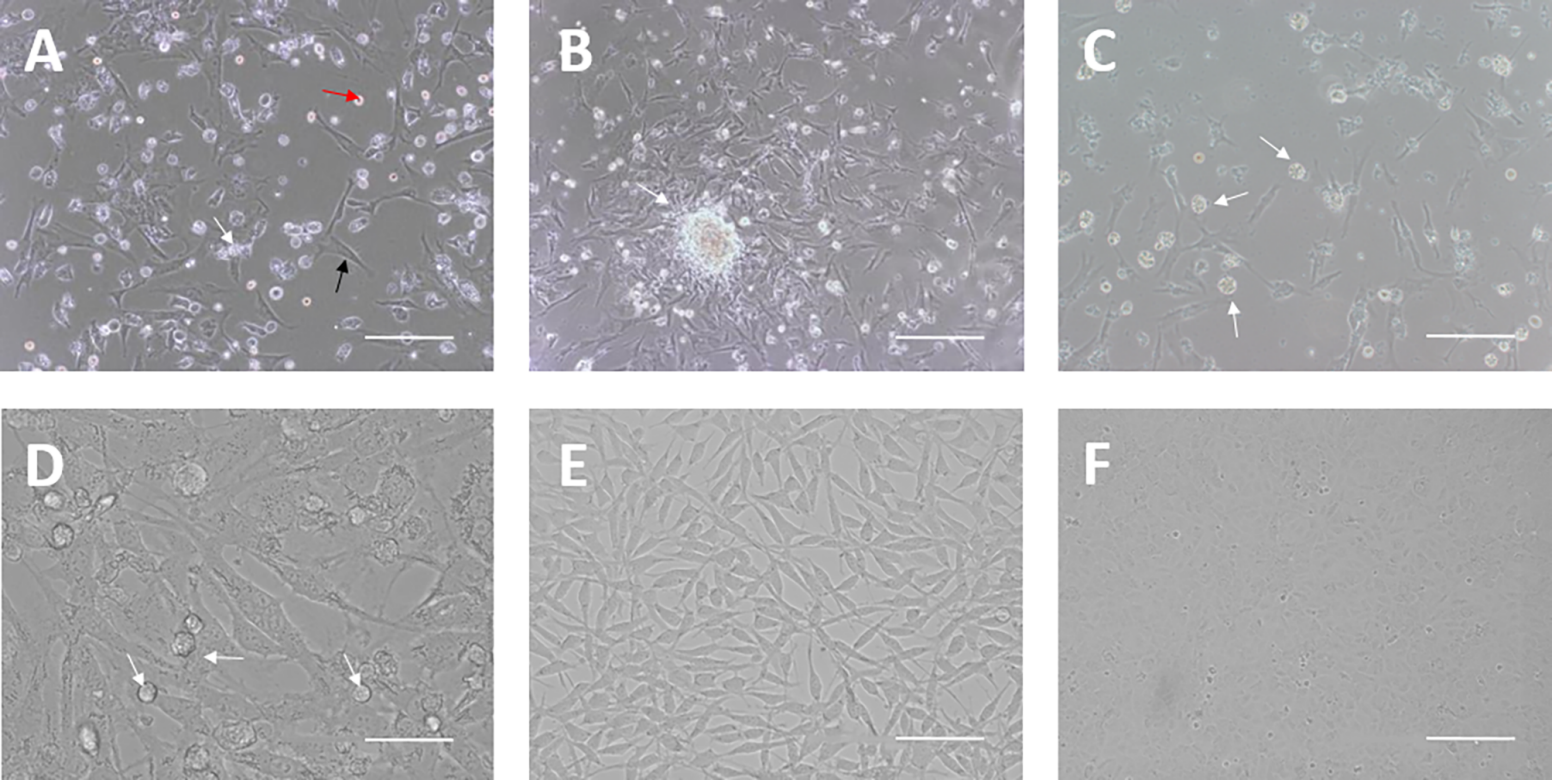
Isolation and culture of chicken PGCs. **(A-D)** Morphology of cultured PGCs. **(A)** PGCs (white arrow) were isolated and primarily cultured with gonadal stroma cells (black arrow) and blood cells (red arrow) from chicken embryos at Stage 27 (Bar = 100 μm). **(B)** PGC colony (white arrow) formation after 3 days of primary culture (Bar = 100 μm). **(C)** Gonadal stroma cells could not support PGCs (white arrows) after 7 days of primary culture (Bar = 100 μm). **(D)** PGCs (white arrows) were then subcultured on feeder layers (mitomycin C treated STO cells) (Bar = 25 μm). **(E)** Morphology of STO cells (Bar = 100 μm). **(F)** Morphology of BRL cells (Bar = 100 μm).

Isolated PGCs were plated together with their own gonadal stroma cells and blood cells from chicken embryos at Stage 27 (Fig 1A). After 4–7 days of culture, most of the blood cells died and PGC colonies became visible on the gonadal stroma cells (Fig 1B). At 10–14 days of growth, gonadal stroma cells could not support the proliferating PGCs and feeder cell layers were needed (Fig 1C). The PGC colonies were dissociated using accutase and then passaged on feeder cells, followed by morphological observation (Fig 1D).

**Fig 2:**
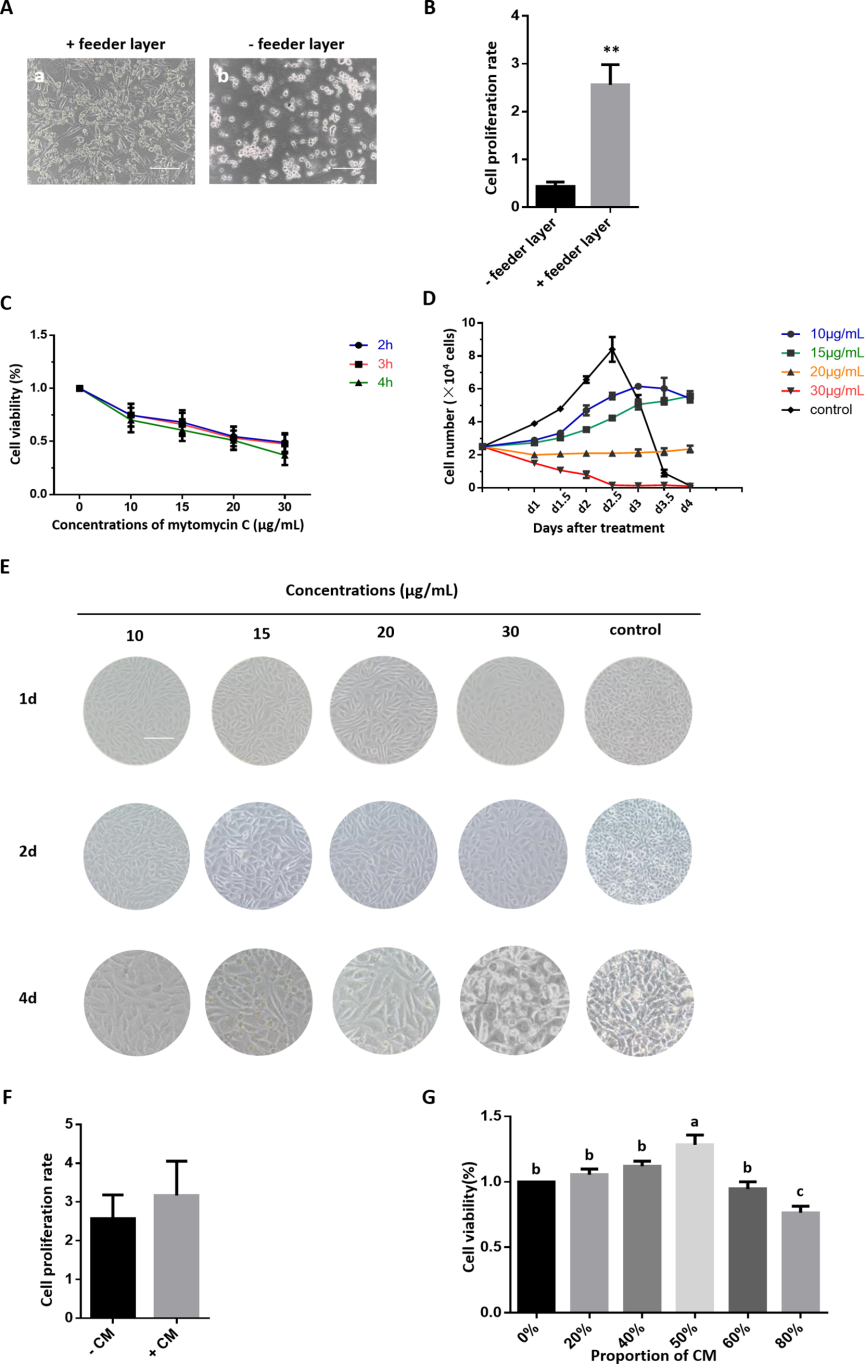
Compound culture system for chicken PGCs. **(A)** Morphology of PGCs in the presence of a feeder layer and at 5 days after bFGF withdrawal (Bar = 100 μm). **(B)** Cell proliferation rate of PGCs in the presence of a feeder layer and at 5 days after feeder layer withdrawal. **(C)** The proliferation and viability rates of mitomycin C treated STO cells detected by a CCK-8 assay; STO cells were treated with various concentrations of mitomycin C (10, 15, 20, 30 μg/mL) for 2, 3, and 4 h, when their confluence reached 60%. **(D)** Growth curve of STO cells treated with different concentrations of mitomycin C. **(E)** Morphology of STO cells treated with different concentrations of mitomycin C. **(F)** Cell proliferation rate of PGCs in the presence of BRL conditioned medium (CM) and at 5 days after CM withdrawal. **(G)** The proliferation and viability rates of PGCs as detected by the CCK-8 assay, the proportion of CM in the medium was 0%, 20%, 40%, 50%, 60%, and 80%, respectively.

Feeder layer cells could support PGC adherence and proliferation, and morphological differentiation of PGCs with or without feeder cells was observable after culturing for 5 days (Fig 2A and 2B). The rate of proliferation in PGCs without the feeder layer was slow with an extremely significant difference, and the culture showed a large amount of cell debris. Mitotically inactivated STO cells (Fig 1E) were chosen as the feeder layer cells. Cell proliferation and viability evaluated using the CCK-8 assay demonstrated that STO cell proliferation and viability was significantly inhibited by mitomycin C in a dose- and time-dependent manner (Fig 2C). As shown in Fig 2D and 2E, the number and density of mitomycin-treated STO cells was markedly smaller compared to the control group. The inhibition of mitomycin-treated STO cells was moderate at 20 μg/mL for 2 h, which was the treatment condition used for the feeder layers in the PGC subculture and follow-on experiments.

BRL cells (Fig 1F) can secrete factors that stimulate the proliferation of PGCs. PGC proliferation and viability was assessed using the CCK-8 assay. As shown in Fig 2F and 2G, BRL conditioned medium could significantly stimulate the proliferation of PGCs, which was inhibited upon excessive addition. The optimal concentration of BRL conditioned medium in the culture medium was found to be 50%, which was then used for PGC subculture and further experiments.

### PGCs maintain high proliferation ability without transformation

**Fig 3:**
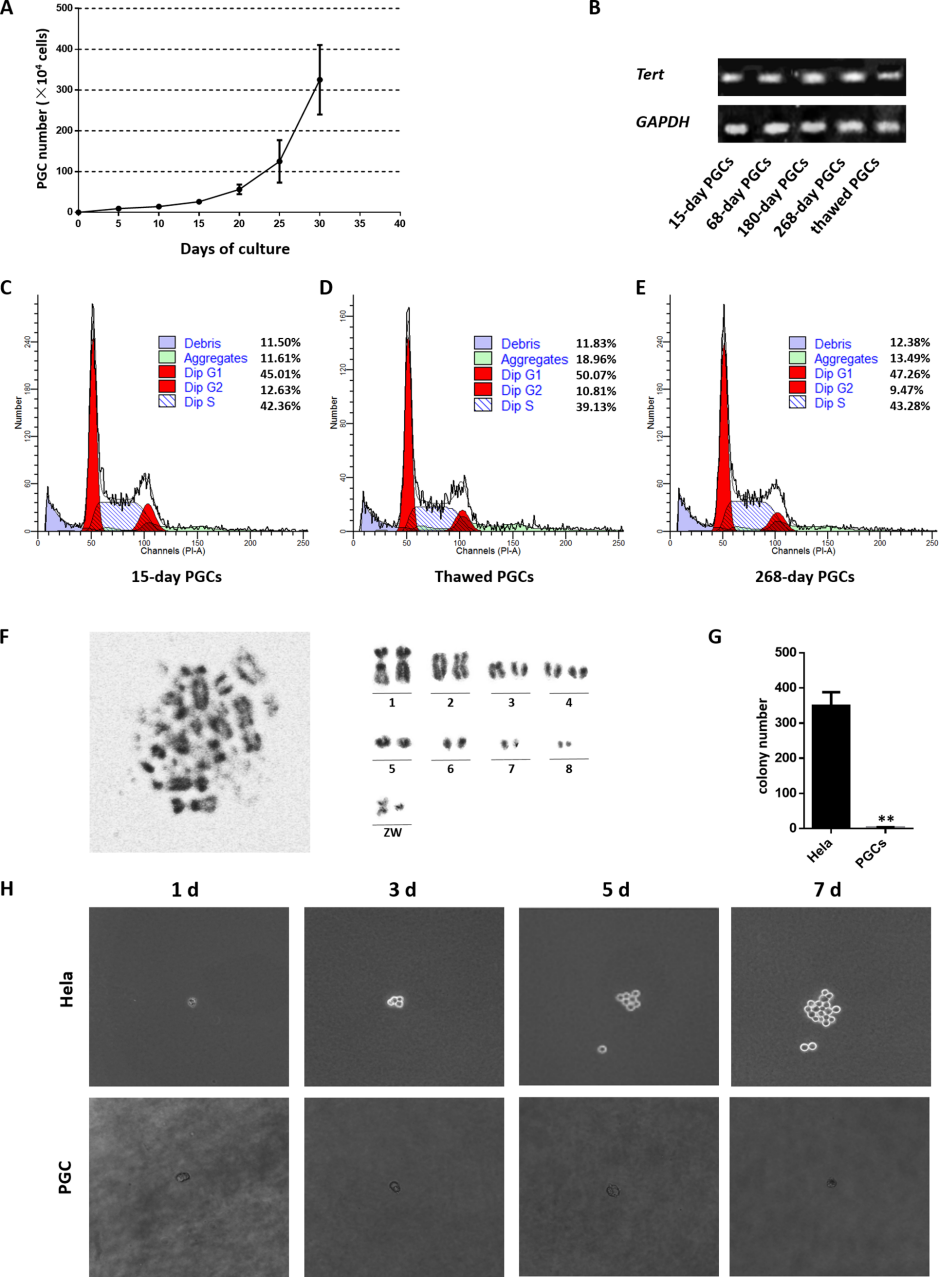
PGC lines maintain high proliferation ability without transformation. **(A)** Growth curve of PGCs isolated and cultured using the culture system above. **(B)** QRT-PCR analysis of *Tert* in cultured PGCs (cultured for 15, 68,180, and 268 days and in thawed cells) **(C-E)** Analysis of cell cycle distribution in fresh PGCs **(C)**, thawed PGCs **(D)**, and long-term cultured PGCs **(E)**. **(F)** Metaphase chromosome (left) and karyotype (right) of chicken PGCs (♀) ZW type; the chromosome karyotype remains diploid. **(G)** The number of clones visible under the stereomicroscope in the soft agar assay after crystal violet staining. **(H)** Colony-forming ability of PGCs. The colony-forming ability of HeLa cells (positive control) (above) and PGCs (below) at day 1, 3, 5, and 7 was assessed by a soft agar assay.

The compound culture system described above supported the growth and proliferation of PGCs for a prolonged time *in vitro*. Isolated PGCs were primarily cultured with their own gonadal stroma cells for a week and were then subcultured in the compound culture system. The growth curve of cultured PGC for the first 30 days is shown in Fig 3A. About 3000 isolated PGCs proliferated to 1 × 10^4^ cells during the first 10 days of culture, and the PGCs then proliferated rapidly and reached approximately 1×10^6^ cells at 20–25 days of culture.

In a long-term culture of 280 days, the PGCs underwent around 91 passages. To examine the telomere activity of PGCs at various culture periods, qRT-PCR was carried out to show that the expression of telomerase reverse transcriptase (*Tert*) was similar across all time points (Fig 3B). At the same time, We still analyzed the cell cycle progression and apoptosis in these cultured PGCs. Flow cytometric analysis showed that the difference in the proliferation of long-term-cultured PGCs and fresh PGCs was not significant and that the cryopreserved cells were slightly inferior, whereas slightly more debris was found in the 268-day-cultured PGCs (Fig 3C, 3D and 3E).

Long-term cultured PGCs maintained a high proliferation rate, and still retained the normal diploid karyotype (Fig 3F). Soft agar colony formation was assessed to examine the colony-forming ability of cultured PGCs. After one week of culture, there were no progressively formed enlarged colonies in PGCs, indicating that PGCs did not undergo transformation and proliferated without tumorigenicity (Fig 3G and 3H).

### Long-term cultured PGCs retain high pluripotency and migration ability

**Fig 4:**
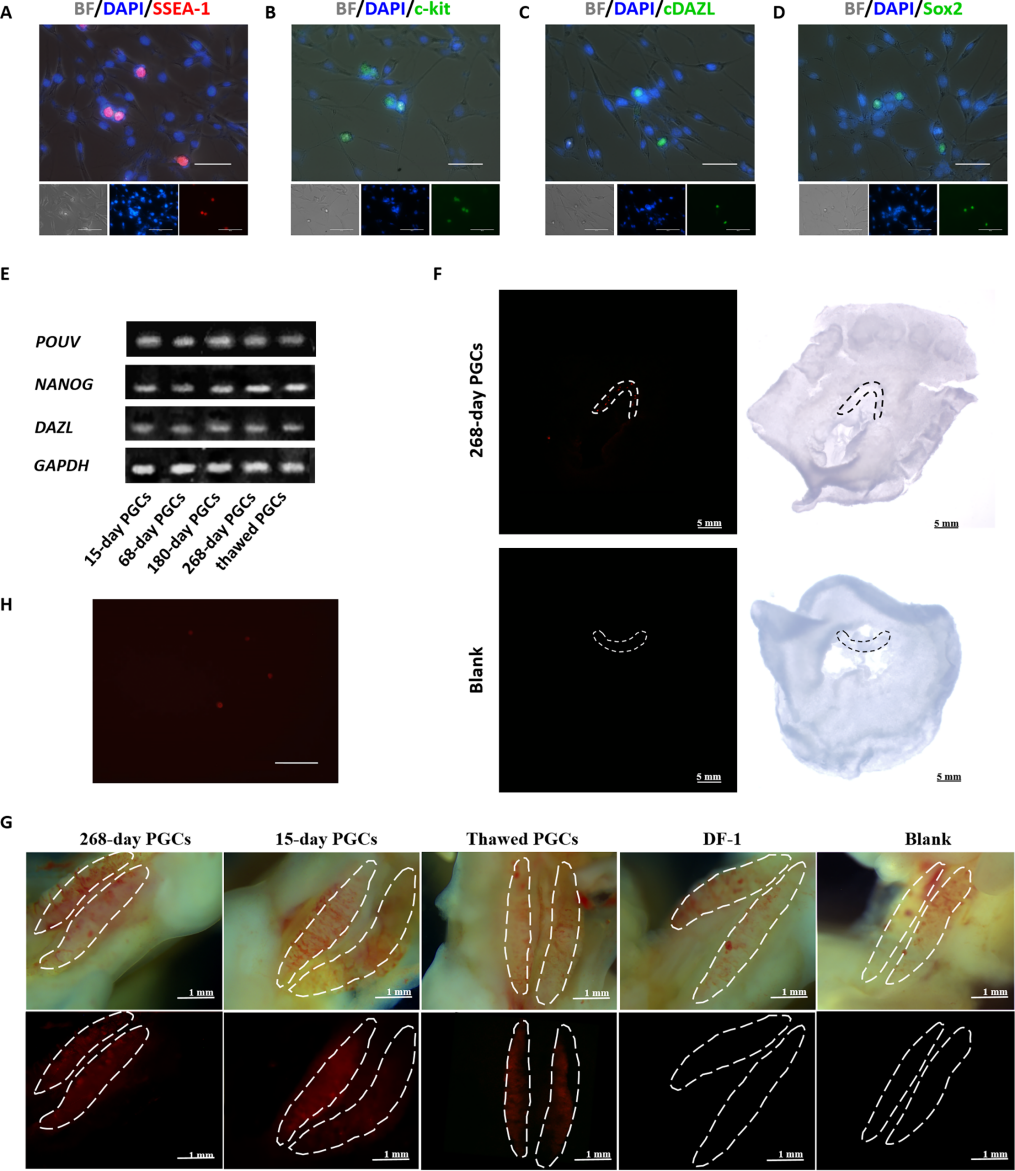
Long-term cultured PGCs maintain high pluripotency and migration ability. **(A-D)** Immunocytochemical analysis of cultured PGCs. PGCs cultured for 180 days were immunostained with antibodies raised against SSEA-1 **(A)**, c-kit **(B)**, cDAZL **(C)**, and Sox2 **(D)**. **(E)** qRT-PCR analysis of *POUV*, *NANOG*, and *DAZL* in cultured PGCs (cultured for 15, 68, 180, and 268 days and in thawed cells) (Bar = 25 μm). **(F)** Migration of cultured PGCs into the germinal crescent. Approximately 3000 PGCs, cultured for 200 days, were labeled with PKH26 and then transferred into the subgerminal cavities of blastoderm embryos. The cells injected were 268-day cultured PGCs (above) and DMEM (below). Labeled cells (red) were detected in the germinal crescent. (Bar = 5 mm) **(G)** Gonadal migration of cultured PGCs. Approximately 3000 cells were labeled with PKH26 and then injected into the blood vessels of recipient embryos at Stage 14–17. From the left to right, the cells injected were 268-day cultured PGCs, 15-day PGCs, thawed PGCs, DF-1, and DMEM. Labeled cells (red) were detected in the embryonic gonad (Bar = 1 mm). **(H)** Cells isolated from the isolated gonad with the PKH26 labeled PGCs (Bar = 100 μm).

The culture system described above supported the pluripotency and migration ability of PGCs for a long time. Indirect immunofluorescence showed that the germline-associated markers SSEA-1, c-kit, cDAZL, and Sox2 were expressed in these cells (Fig 4A, 4B, 4C and 4D). The results of qRT-PCR showed that the PGCs expressed the stage specific genes *POUV* and *DAZL*, and the stem cell specific gene *NANOG* (Fig 4E). *In vivo* PGC migration showed that the cells retained the ability to migrate to the germinal crescent (Fig 4F) and gonads (Fig 4G) normally, and that the injected cells could still be isolated and observed *in vitro* (Fig 4H).

### Long-term cultured PGCs retain their differentiation ability *in vitro*

**Fig 5:**
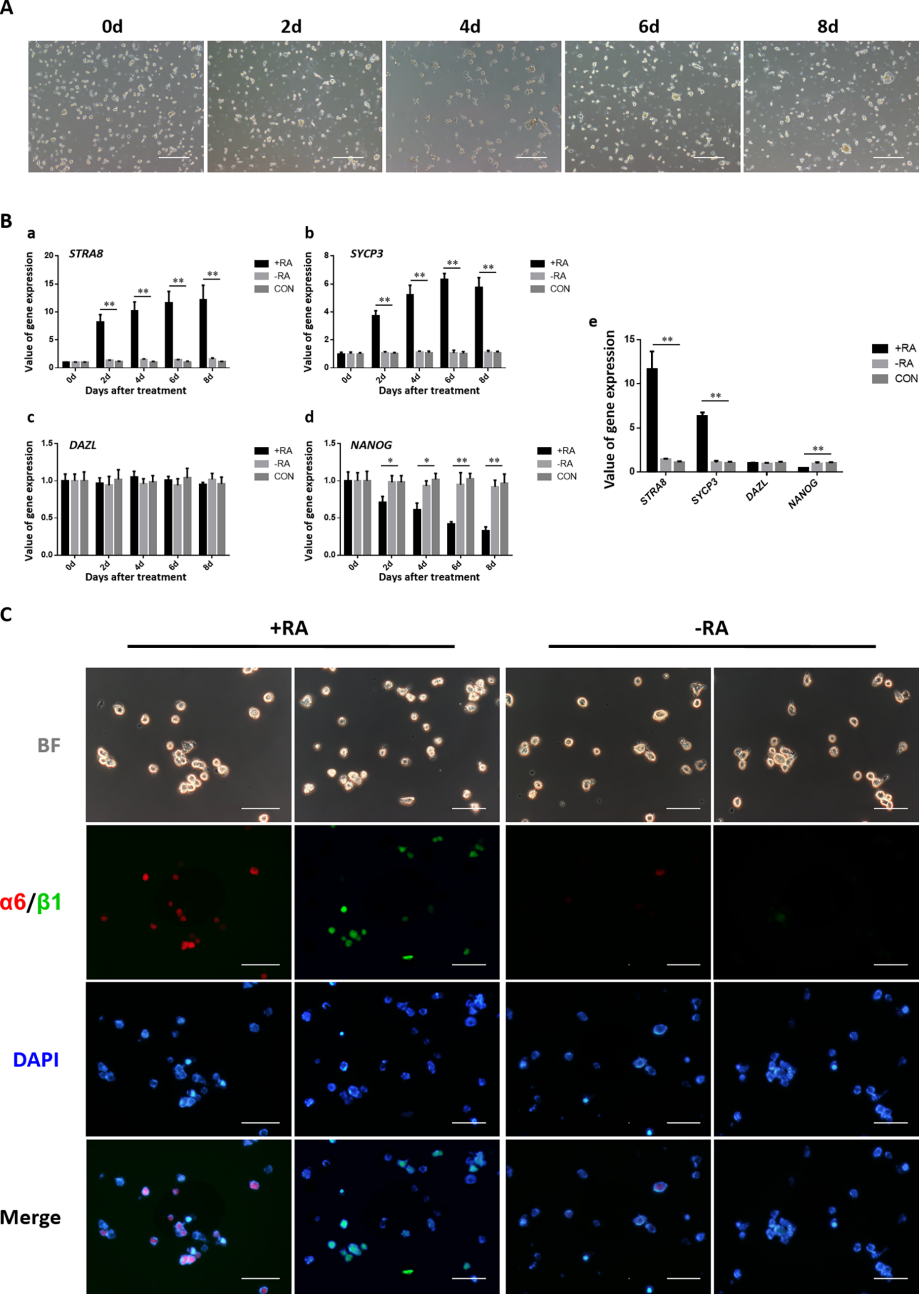
Long-term cultured PGCs retain their differentiation ability *in vitro*. **(A)** Morphological changes in PGCs upon RA induction. The long-term cultured PGCs were treated with 10 μmol/L RA for 8 days and showed differentiation into presumptive SSC-like cells (Bar = 100 μm). **(B)** Quantitative RT-PCR was performed to verify the related genes in the presence or absence of RA induction for 0, 2, 4, 6, and 8 days, respectively, using SSC-specific (*STRA8* **(a)**, *SYCP3* **(b)**), germness-related (*DAZL* **(c)**), and stemness-related (*NANOG* **(d)**) genes. The gene expression values at day 6 are shown in **(e)**. **(C)** Immunocytochemistry for SSC marker genes *integrinα6* (Red) and *integrinβ1* (Green) at day 8 in the presence or absence of RA induction (Bar = 25 μm).

The compound culture system supported the differentiation of PGCs *in vitro*. RA induced differentiation and presumptive SSC-like cell formation in the cultured PGCs. The growth state of cultured PGCs in the induction medium at 0, 2, 4, 6, and 8 days was observed under an inverted microscope (Fig 5A). QRT-PCR was used to determine the expression of the meiotic promoter gene *STRA8*, meiosis-specific gene *SYCP3*, the germ cell specific gene *DAZL*, and stem cell specific gene *NANOG* (Fig 5B). The results showed that the expression of *STRA8* as well as *SYCP3* in the +RA condition was significantly upregulated. There was no significant difference in *DAZL* expression under the influence of RA. Expression of the PGC-specific marker *NANOG*, was significantly reduced to 0.33 in a week in the treatment of RA. These results indicate that RA treatment to PGCs can help induce meiosis. Simultaneously, indirect immunofluorescence after 8 days of RA treatment, showed that the expression of *integrinα6* and *integrinβ1*, as meiotic germ cell markers, was increased significantly (Fig 5C).

**Fig 6:**
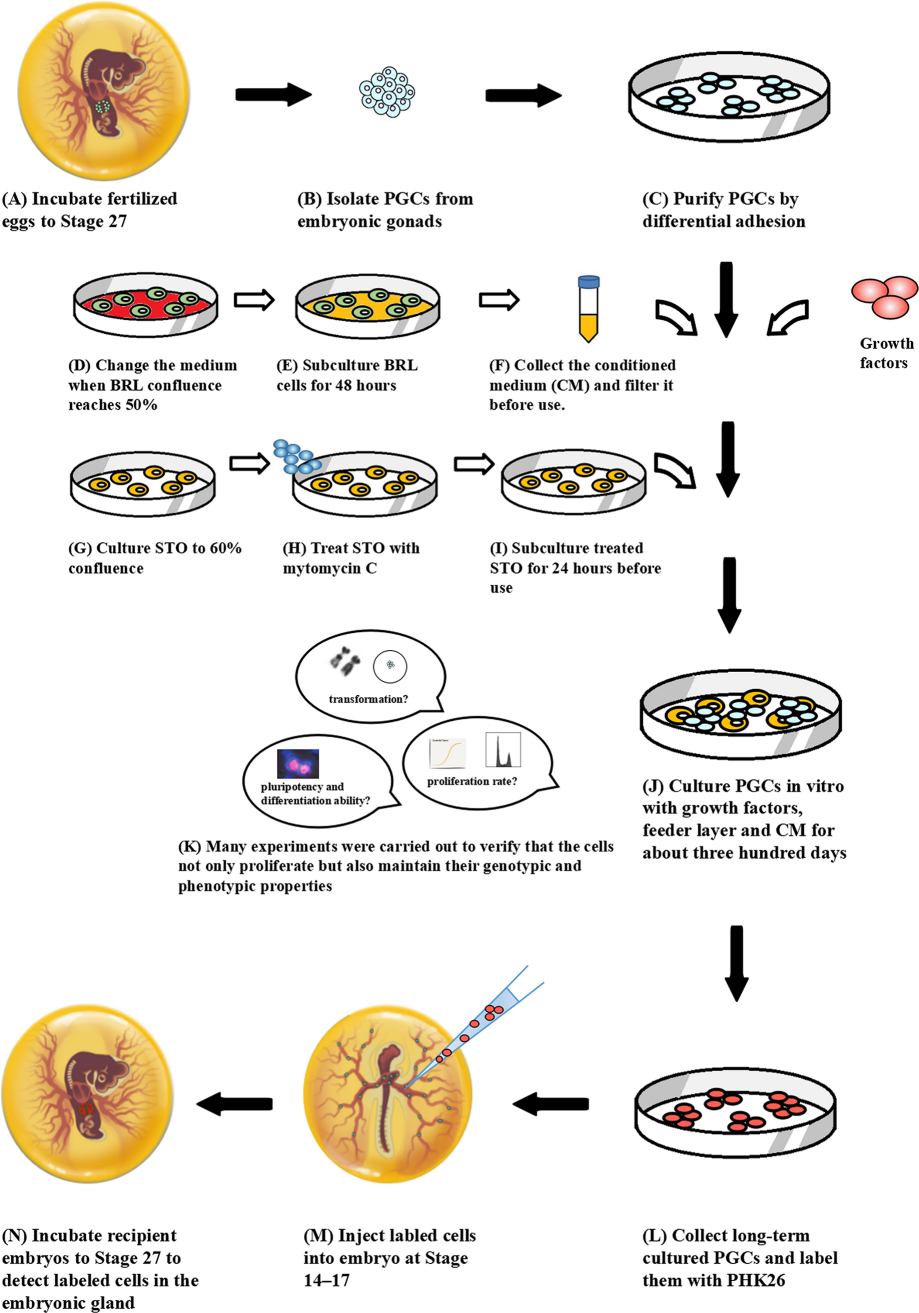
The technical scheme for chicken PGCs long-term culture in vitro and preliminary establishment of cell lines.

This PGCs technology (Fig 6) has been successfully retained high pluripotency of PGCs *in vitro* for about three hundred days without any differentiation and transformation. All the above demonstrated that we have developed a robust compound culture system and have done the preliminary establishment of chicken primordial germ cell lines.

## Discussion

We have developed a stable compound culture system for PGC isolation, long-term culture, cryopreservation, and thawing, and successfully maintained cultured PGCs in the proliferation stage *in vitro* for about three hundred days without differentiation. Since Dubois reported them in 1969 [13], several studies have been conducted on chicken PGCs. Allioli et al. (1994) compared three methods of isolating gonadal PGCs and cultured PGCs *in vitro* for a short time in DMEM/F12 medium containing 10% fetal calf serum. Park and Han supplemented hSCF, LIF, bFGF, IL-11, and IGF-I in culture medium and finally maintained the cells for up to two months [14]. With the rapid developments in this field, the maintenance time for PGCs *in vitro* has been extended from three days to several months or even more [10, 15, 16]. It has been demonstrated that elements affecting the *in vitro* PGC culture include serum, growth factors, feeder layer, the micro-environment, and the state of the PGCs themselves.

Since 1992, some essential growth factors that maintain PGC survival, proliferation and growth *in vitro* have been found. The specific growth factors LIF, bFGF, and hSCF are reportedly required for the *in vitro* culture of PGCs. LIF is a differentiation inhibitory factor that can inhibit embryonic stem cell differentiation and promote cell proliferation to maintain the pluripotency of cells, and can be obtained from BRL conditioned medium. bFGF is a potent mitogen of the PGC-feeder layer system that regulates PGC proliferation and survival. Meanwhile, hSCF can enhance the mitotic activity and motility of PGCs and inhibit apoptosis to maintain their survival. In mice and humans, these three growth factors and STO feeder layers are essential for the establishment of embryonic germ cell lines [5, 17, 18]. Choi et al. reported that PGCs can maintain their proliferation and survival rates without differentiation or de-differentiation in culture medium supplemented with LIF, bFGF, and hSCF [16]. We found that culture of PGCs in the absence of any of these factors resulted in cell differentiation to various types over several days.

Van de Lavoir et al. reported that PGCs can be maintained and proliferated with STO or BRL feeder cells and BRL-conditioned medium [10]. As described by Choi et al., feeder layer cells can secrete growth factors and support adherent growth to provide a suitable growth environment for PGCs and inhibit their differentiation [19]. As reported in a previous study, gonad PGCs were spherical with a diameter of about 12–17 μm with microvilli on the surface [20]. When the PGCs adhered to a feeder layer, the majority of PGCs produced small pseudopodia similar to their condition *in vivo* [21]. We tried using a feeder layer-free culture system and found a large number of cell debris in 5 days of culture. The difference in the proliferation rate between the PGCs in two culture systems was extremely significant, indicating that the feeder layer is essential to PGCs in the absence of any other treatment. On the other hand, the quality of the feeder layer plays a significance role and an inappropriate feeder layer will have a negative effect. The time and dose of mitomycin C treatment and the proper seeding density both affect the growth of PGCs. A strong growth rate and high-density feeder layer will inhibit the target cells, whereas excessive treatment and low-density fibroblasts cannot form sufficient growth factors, and on the contrary, may even destroy the chromosomal structure of PGCs. In the present study, STO cells were inactivated with 20 μg/mL mitomycin C for 2 h when their confluence reached about 60%, then washed with PBS for 5–8 times after treatment, and subcultured in complete medium for over 24 h to ensure complete removal of mitomycin C. Furthermore, BRL-conditioned medium contains cell secretions like LIF and simulates the microenvironment of numerous cells, which can stimulate proliferation, inhibit differentiation, and help PGCs pass through the low growth rate and high differentiation period quickly [10]. Hence, 50% BRL-conditioned medium was supplemented with the factor-containing medium preconditioned on BRL, collected, and stored at -80°C before use in case the factors weakened. Meanwhile, we also tried using a culture system without conditioned medium and observed that the proliferation rate of PGCs was decreased slightly. Therefore, we speculated that the major role of conditioned medium might be to provide growth factors, and can be replaced when essential growth factors are supplied at sufficient concentrations to support the *in vitro* proliferation of PGCs.

Significant progress has been made in the culture systems for PGCs, and the maintenance time of PGCs *in vitro* has been extended from three days to several months or even more [10, 22, 23]. The low proliferation rate and high tendency for differentiation in the cultured PGCs make then an incompatible method for further manipulations. Therefore, methodological improvements were needed to maintain the undifferentiated state and efficient proliferation in PGCs. In this study, we have attempted to adjust a variety of conditions in the culture system such as culturing our PGCs without STO feeders in gelatin-coated dishes to prevent cell attachment; under these conditions, the PGCs reached a proliferation plateau and adhered for differentiation within several weeks. Ultimately an appropriate method for the culture of chicken gonad PGCs was developed, which can maintain proliferation *in vitro* for about three hundred days without differentiation, with a much longer and greater stability than that reported previously. In primary culture, about 3000 PGCs were isolated and proliferated 40-times during the first 10 days of culture; these PGCs were then transferred to the compound culture system, where they showed a striking proliferation rate requiring their passage after every 3 days and changing their culture medium almost every day.

Within this culture period, we performed many experiments to verify that the cells proliferate quantitatively as well as maintain their cellular properties in both their genotype and phenotype. We examined the expression of several germline-associated markers such as cDAZL, c-kit, and Sox2 [24–26], and PGC-specific markers such as SSEA-1. These efficient gametogenesis genes were expressed without any significant differences throughout the long-term culture and cryopreservation. The result of qRT-PCR analysis led to the same conclusion. Except the markers related to the PGC phenotype, the expression of *Tert* suggested conservation of their original expression level, which was considered as telomerase activity and indicated the potential for unlimited cell proliferation [27]. The unique migration activity of chicken PGCs is a key characteristic differing from other species. In the present study, cultured PGCs moved actively to the germinal crescent and gonads similar toendogenous PGCs, suggesting conservation of their germline competency [18]. Furthermore, the PGCs maintained normal diploid karyotype without transformation and tumorigenicity, and the formation of SSC-like cells *in vitro* showed that the PGCs retain the potentially capable of normal differentiation. All the results indicated that our culture conditions allow the maintenance of an original phenotype as *in vivo*.

Recent studies have shown that RA plays a decisive role in embryonic germ cells entering meiosis. The RA signal initiates meiosis in the ovaries of mice by stimulating the expression of *Stra8* [28]. The RA in mouse embryonic testicular germ cells is degraded by the RA metabolic enzyme *Cyp26b1* expressed by testis somatic cells so that the germ cells cannot initiate meiosis. At maturity, *Cyp26b1* expression is decreased, and the RA signal is activated. Studies have shown that RA determines the beginning of meiosis. In this regard, we attempted to induce PGC differentiation *in vitro* using RA and observed the formation of presumptive SSC-like cells; expression of SSC-specific markers *integrinα6*and *integrinβ1* was significantly increased after RA induction. These results showed that the long-term cultured PGCs retain their ability of differentiation.

We concluded that the compound culture system developed is a practical and efficient method for developing chicken PGC lines. PGCs cultured in this system are pluripotent and retain their unique ability to migrate without transformation, suggesting that they can be used as carriers in transgenic bioreactors. The mechanism of cell immortalization has been explored initially and the foundation for the follow-up establishment of PGC lines has been laid. Although most species use ES for stem cell manipulation, the special developmental pattern of chickens makes it difficult to obtain chicken ES compared to PGCs. Meanwhile, ES cells contribute to somatic tissues whereas PGCs directly contribute to the germ line, which is more efficient and stable. Our results suggest that the unlimited expansion of PGCs is feasible, which can contribute to the preservation and propagation of avian genetic resources and even to the generation of transgenic bioreactors and avian germline manipulation.

## Supporting information

S1 Table Primers used for qRT-PCR analysis. (DOCX)

S2 Table Culture medium for PGCs induction *in vitro*. (DOCX)

## References

[1] Nieuwkoop PD and Sutasurya LA. Primordial germ cells in the chordates: embryogenesis and phylogenesis CUP Archive; 1980.

[2] Hamburger V and Hamilton HL. A series of normal stages in the development of the chick embryo. J Morphol. 1951; 881:49–92. doi: 10.1002/JMOR.1050880104 24539719

[3] Naito M, Harumi T and Kuwana T. Long-term culture of chicken primordial germ cells isolated from embryonic blood and production of germline chimaeric chickens. Anim Reprod Sci. 2015; 153:50–61. doi: 10.1016/j.anireprosci.2014.12.003 25578502 10.1016/j.anireprosci.2014.12.003

[4] Leighton PA, van de Lavoir MC, Diamond JH, Xia C, and Etches RJ. Genetic modification of primordial germ cells by gene trapping, gene targeting, and phiC31 integrase. Mol Reprod Dev. 2008; 75:1163–1175. doi: 10.1002/mrd.20859 18213680 10.1002/mrd.20859

[5] Nakamura Y, Usui F, Miyahara D, Mori T, Ono T, Takeda K, et al Efficient system for preservation and regeneration of genetic resources in chicken: concurrent storage of primordial germ cells and live animals from early embryos of a rare indigenous fowl (Gifujidori). Reprod. Fertil. Dev. 2010; 22:1237–1246. doi: 10.1071/RD10056 20883649 10.1071/RD10056

[6] Macdonald J, Taylor L, Sherman A, Kawakami K, Takahashi Y, Sang HM, et al Efficient genetic modification and germ-line transmission of primordial germ cells using piggyBac and Tol2 transposons. Proc. Natl Acad. Sci. USA, 2012; 109:E1466–E1472. doi: 10.1073/pnas.1118715109 22586100 10.1073/pnas.1118715109 PMC3384192

[7] Park TS, and Han JY. piggyBac transposition into primordial germ cells is an efficient tool for transgenesis in chickens. Proc. Natl Acad. Sci. USA. 2012; 109:9337–9341. doi: 10.1073/pnas.1203823109 22645326 10.1073/pnas.1203823109 PMC3386051

[8] Liu C, Khazanehdari KA, Baskar V, Saleem S, Kinne J, Wernery U, et al Production of chicken progeny *(Gallus gallus domesticus)* from interspecies germline chimeric duck *(Anas domesticus)* by primordial germ cell transfer. Biol. Reprod. 2012; 86:101 doi: 10.1095/biolreprod.111.094409 22190706 10.1095/biolreprod.111.094409

[9] Song Y, Duraisamy S, Ali J, Kizhakkayil J, Jacob VD, Mohammed MA, et al Characteristics of long-term cultures of avian primordial germ cells and gonocytes. Biol. Reprod. 2014; 90:15 doi: 10.1095/biolreprod.113.113381 24337317 10.1095/biolreprod.113.113381

[10] van de Lavoir MC, Diamond JH, Leighton PA, Mather-Love C, Heyer BS, Bradshaw R, et al Germline transmission of genetically modified primordial germ cells. Nature. 2006; 441 7094:766–769. doi: 10.1038/NATURE04831 16760981 10.1038/nature04831

[11] Han JY. Germ cells and transgenesis in chickens. Comp Immunol Microbiol Infect Dis. 2009; 32(2):61–80. doi: 10.1016/j.cimid.2007.11.010 18249442 10.1016/j.cimid.2007.11.010

[12] Eyal-Giladi H, Kochav S. From cleavage to primitive streak formation: a complementary normal table and a new look at the first stages of the development of the chick. I. General morphology. Dev Biol. 1976; 49:321–337. doi: 10.1016/0012-1606(76)90178-0 944662 10.1016/0012-1606(76)90178-0

[13] Dubois R. Localization of primordial germinal cells on the non-incubated chicken germ layers. C R Acad Sci Hebd Seances Acad Sci D. 1969; 2692:205–208. 4982813

[14] Park TS, Han JY. Derivation and characterization of pluripotent embryonic germ cells in chicken. Mol Reprod Dev. 2000; 56:475–482. doi: 10.1002/1098-2795(200008)56:4<475::AID-MRD5>3.0.CO;2-M 10911397 10.1002/1098-2795(200008)56:4<475::AID-MRD5>3.0.CO;2-M

[15] Macdonald J, Glover JD, Taylor L, Sang HM, McGrew MJ. Characterisation and germline transmission of cultured avian primordial germ cells. PLoS One. 2010; 5(11):e15518 doi: 10.1371/journal.pone.0015518 21124737 10.1371/journal.pone.0015518 PMC2993963

[16] Shiue YL, Tailiu JJ, Liou JF, Lu HT, Tai C, Shiau JW, et al Establishment of the long-term in vitro culture system for chicken primordial germ cells. Reprod Domest Anim. 2009; 44(1):55–61. doi: 10.1111/j.1439-0531.2007.00990.x 18484956 10.1111/j.1439-0531.2007.00990.x

[17] Kim JN, Park TS, Park SH, Park KJ, Kim TM, Lee SK, et al Migration and proliferation of intact and genetically modified primordial germ cells and the generation of a transgenic chicken. Biol Reprod. 2010; 82 (2):257–262. doi: 10.1095/biolreprod.109.079723 19710509 10.1095/biolreprod.109.079723

[18] Lee HJ, Lee HC, Kim YM, Hwang YS, Park YH, Park TS, et al Site-specific recombination in the chicken genome using Flipase recombinase-mediated cassette exchange. FASEB J. 2016; 30(2):555–563. doi: 10.1096/fj.15-274712 26443821 10.1096/fj.15-274712

[19] Choi JW, Kim S, Kim TM, Kim YM, Seo HW, Park TS, et al Basic fibroblast growth factor activates MEK/ERK cell signaling pathway and stimulates the proliferation of chicken primordial germ cells. PLoS One. 2010; 5(9):e12968 doi: 10.1371/journal.pone.0012968 20886037 10.1371/journal.pone.0012968 PMC2944891

[20] England MA, Matsumura G. Primordial germ cells in the primitive streak stages chick embryo as studied by scanning electron microscopy. J Anat. 1993; 183:67–73. 8270477 PMC1259854

[21] Kuwana T, Miyayama Y, Kajiwara Y, and Fujimoto T. Behavior of chick primordial germ cells moving toward gonadal primordium in vitro: scanning electron microscopic study. Anat Rec. 1987; 219:164–170. doi: 10.1002/ar.1092190209 3688470 10.1002/ar.1092190209

[22] Kito G, Aramaki S, Tanaka K, Soh T, Yamauchi N, and Hattori MA. Temporal and spatial differential expression of chicken germline-specific proteins cDAZL, CDH and CVH during gametogenesis. J. Reprod. Dev. 2010; 56:341–346. doi: 10.1262/JRD.09-218A 20332590 10.1262/jrd.09-218a

[23] Cañón S, Herranz C, and Manzanares M. Germ cell restricted expression of chick Nanog. Dev. Dyn. 2006; 235:2889–2894. doi: 10.1002/dvdy.20927 16921504 10.1002/dvdy.20927

[24] Naeemipour M, Dehghani H, Bassami M, and Bahrami A. Expression dynamics of pluripotency genes in chicken primordial germ cells before and after colonization of the genital ridges. Mol. Reprod. Dev. 2013; 80:849–861. doi: 10.1002/mrd.22216 23877993 10.1002/mrd.22216

[25] Bodnar AG, Wright WE. Extension of life-span by introduction of telomerase into normal human cells. Science. 1998; 279:349–352. doi: 10.1126/SCIENCE.279.5349.349 9454332 10.1126/science.279.5349.349

[26] Xu L, Chang G, Ma T, Wang H, Chen J, Li Z, et al *Piwil1* mediates meiosis during spermatogenesis in chicken. Ani repro sci. 2016; 166:99–108. doi: 10.1016/j.anireprosci.2016.01.008 26811260 10.1016/j.anireprosci.2016.01.008

[27] Kapoor A. Retinoic Acid-Elicited RARα/RXRα Signaling attenuates a beta production by directly inhibiting gamma-Secretase-Mediated cleavage of amyloid precursor protein. Acs Chemical Neuroscience. 2013; 4(7):1093–100. doi: 10.1021/cn400039s 23530929 10.1021/cn400039s PMC3715835

[28] Lavial F, Acloque H, Bertocchini F, MacLeod DJ, Boast S, Bachelard E, et al The Oct4 homologue PouV and Nanog regulate pluripotency in chicken embryonic stem cells. Development. 2007; 134:3549–3563. doi: 10.1242/dev.006569 17827181 10.1242/dev.006569

