# Supplementary material for: Long-term in vitro culture and preliminary establishment of chicken primordial germ cell lines
Source: PLoS One. 2018 Apr 30;13(4):e0196459. doi: 10.1371/journal.pone.0196459 (PMC5927411; doi:10.1371/journal.pone.0196459)
Supplement: S1 Table — (DOCX) [file pone.0196459.s001.docx]

**S1** **Table** Primers used for qRT-PCR analysis.

| Gene | Primer sequence (5’-3’) | Reference |
| --- | --- | --- |
| *NANOG* | F: CAGCAGACCTCTCCTTGACC  R: AAGCCCTCATCCTCCACAGC | Choi et al. ^[19]^ |
| *POUV* | F: GTTGTCCGGGTCTGGTTCT  R:GTGGAAAGGTGGCATGTAGAC | Lavial et al. ^[28]^ |
| *DAZL* | F: CGTCAACAACCTGCCAAGGA  R:TTCTTTGCTCCCCAGGAACC | Choi et al. ^[19]^ |
| *Tert* | F: GTCAGAGCGAAGTCATCACAAGAAT  R:TGGCAAAACTCTGAAGTGACAAC | Swanberg et al. ^[29]^ |
| *GAPDH* | F: CACAGCCACACAGAAGACGG  R:CCATCAAGTCCACAACACGG | Choi et al. ^[19]^ |
| *STRA8* | F: GTGAGGGACAGTGGAGGTAA  R: CAGAAATGCCGCTTGTAAAT | Kapoor^[27]^ |
| *SYCP3* | F: CTGTATTTCAGCAGTGGGATG  R: TGCGAAGTTCATTTTGTGC | Pramod et al. ^[30]^ |

**References**

1. Swanberg SE, Payne WS, Hunt HD, Dodgson JB, Delany ME. Telomerase activity and differential expression of telomerase genes and c-myc in chicken cells in vitro. Dev Dyn. 2004; 231:14–21. doi: 10.1002/DVDY.20149
2. Pramod RK, Lee BR, Kim YM, Lee HJ, Park YH, Ono T, et al. Isolation, Characterization, and In Vitro Culturing of Spermatogonial Stem Cells in Japanese Quail (Coturnix japonica). Stem Cells & Development. 2017; 26(1):60. doi: 10.1089/scd.2016.0129
