# Supplementary material for: Long-term in vitro culture and preliminary establishment of chicken primordial germ cell lines
Source: PLoS One. 2018 Apr 30;13(4):e0196459. doi: 10.1371/journal.pone.0196459 (PMC5927411; doi:10.1371/journal.pone.0196459)
Supplement: S2 Table — (DOCX) [file pone.0196459.s002.docx]

**S2** **Table** Culture medium for PGC induction *in vitro*.

| Group | Complete medium  (factors withdrawn) | RA | Factors (LIF, bFGF, hSCF) |
| --- | --- | --- | --- |
| +RA | + | + | - |
| -RA | + | - | - |
| CON | + | - | + |
